# *PRKACA*: the catalytic subunit of protein kinase A and adrenocortical tumors

**DOI:** 10.3389/fcell.2015.00026

**Published:** 2015-05-20

**Authors:** Annabel S. Berthon, Eva Szarek, Constantine A. Stratakis

**Affiliations:** Section on Endocrinology and Genetics, Program on Developmental Endocrinology and Genetics and Pediatric Endocrinology Inter-Institute Training Program, Eunice Kennedy Shriver National Institute of Child Health and Human Development, National Institutes of HealthBethesda, MD, USA

**Keywords:** adrenal cortex, adenoma, *PRKACA*, PKA, Cushing syndrome

## Abstract

Cyclic-AMP (cAMP)-dependent protein kinase (PKA) is the main effector of cAMP signaling in all tissues. Inactivating mutations of the *PRKAR1A* gene, coding for the type 1A regulatory subunit of PKA, are responsible for Carney complex and primary pigmented nodular adrenocortical disease (PPNAD). *PRKAR1A* inactivation and PKA dysregulation have been implicated in various types of adrenocortical pathologies associated with ACTH-independent Cushing syndrome (AICS) from PPNAD to adrenocortical adenomas and cancer, and other forms of bilateral adrenocortical hyperplasias (BAH). More recently, mutations of *PRKACA*, the gene coding for the catalytic subunit C alpha (Cα), were also identified in the pathogenesis of adrenocortical tumors. *PRKACA* copy number gain was found in the germline of several patients with cortisol-producing BAH, whereas the somatic Leu206Arg (c.617A>C) recurrent *PRKACA* mutation was found in as many as half of all adrenocortical adenomas associated with AICS. *In vitro* analysis demonstrated that this mutation led to constitutive Cα activity, unregulated by its main partners, the PKA regulatory subunits. In this review, we summarize the current understanding of the involvement of *PRKACA* in adrenocortical tumorigenesis, and our understanding of PKA's role in adrenocortical lesions. We also discuss potential therapeutic advances that can be made through targeting of *PRKACA* and the PKA pathway.

## Introduction

The adrenal cortex is divided into three concentric zones: the outermost zone named *zona glomerulosa*, the centrally located *zona fasciculata* and the innermost, *zona reticularis* involved in the production of mineralocorticoids, glucocorticoids, and androgens, respectively (Blake et al., 2008; Mcnicol, 2013). Thus, adrenal dysfunction leads to several hormonal syndromes due to the hypo- or hyper-secretion of one or more adrenal hormones.

In this review, we focus on Cushing's syndrome (CS) resulting from overproduction of cortisol from adrenocortical tumors (ACT). CS leads to central obesity and metabolic abnormalities and several other manifestations including moon face, buffalo hump, striae, and opportunistic infections (Newell-Price et al., 2006). Severe and prolonged hypercortisolism could lead to increased morbidity and mortality, due to sepsis, cardiovascular, and other complications (Plotz et al., 1952; Arnaldi et al., 2012). Hypersecretion of cortisol can be due to either an excess of pituitary or ectopic adrenocorticotropin hormone (ACTH) secretion or adrenocortical tumors (ACT) secreting cortisol autonomously; the latter form of CS is known as “ACTH-independent CS” (AICS).

Cortisol-producing ACTs include bilateral adrenocortical hyperplasias (BAH), adrenocortical adenomas (ACA) and cancer (ACC). BAHs account for 10–15% of AICS and are classified in two subtypes: macronodular (nodules >1 cm) and micronodular (nodules <1 cm) (Lacroix, 2009; Duan et al., 2014). Macronodular hyperplasia, previously known as massive macronodular adrenocortical disease (MMAD) or ACTH-independent macronodular adrenocortical hyperplasia (AIMAH), has been recently renamed as primary macronodular hyperplasia (PMAH) after the discovery of intra-adrenal ACTH production (Lacroix, 2013; Louiset et al., 2013). PMAH is typically diagnosed in the fifth and sixth decade of life; subclinical CS is common in this disease (Duan et al., 2014), despite the fact that macroscopically, the adrenal glands are massively enlarged with a combined weight reach from 60 to 200 g.

Micronodular hyperplasias include a pigmented form named primary pigmented nodular adrenocortical disease (PPNAD), which is typically diagnosed at a younger age. PPNAD is the most common endocrine lesion of Carney complex (CNC), occurring in more than 60% of CNC patients (Almeida and Stratakis, 2010). Grossly, PPNAD is associated with normal to slightly enlarged adrenal glands (4.3–17 g) with a large number of yellow to brown-black micro-nodules (0.1–0.3 mm in size) due to lipofuscin accumulation (responsible for the pigmentation) (Stratakis and Boikos, 2007; Stratakis, 2008).

Unilateral ACTs, ACAs account for 90% of adrenal CS (Newell-Price et al., 2006; Bertagna et al., 2009). Clinically, these tumors arise at any age, with a slight female predominance. The presentation ranges from subclinical to overt CS. Macroscopically, the average ACA ranges in size from 1.5 cm to 6 cm and weighs between 10 and 40 g. In contrast to ACAs, ACCs are rare and account for few cases of adrenal CS (Wajchenberg et al., 2000). They arise sporadically, mostly around the fourth and fifth decade of life; ACCs typically weigh more than 100 g, tend to be adherent to other tissues, or invade adjacent structures.

## cAMP signaling, *PRKAR1A* defects, and adrenocortical tumors

**Figure 1:**
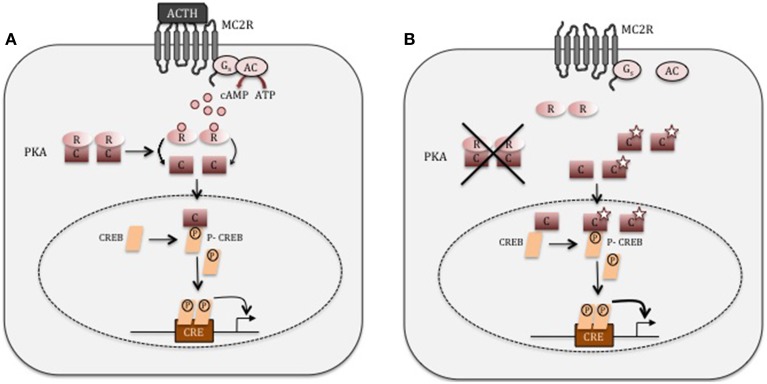
**cAMP signaling. (A)** In normal adrenocortical cells, ACTH binds to its G-coupled receptor, MC2R. This leads to the activation of adenylate cyclase (AC), which convert ATP into cAMP. cAMP then binds the regulatory (R) subunit of PKA, inducing the release of the catalytic subunit (C). The catalytic subunit phosphorylates its downstream target such as CREB, which in turn induces the expression of genes involved in cortisol synthesis. **(B)** In adrenocortical adenoma cells producing cortisol autonomously with *PRKACA* mutations (star), the catalytic (C) subunit of PKA is unable to interact with the regulatory subunit (R). The unregulated *PRKACA* may now mediate its serine-threonine kinase activity without any restrains.

In normal physiology, cAMP signaling plays an essential role in the regulation of cortisol secretion under the control of the hypothalamic-pituitary-adrenal axis (Figure 1). Hypothalamic corticotropin-releasing hormone (CRH) secretion stimulates ACTH secretion from the pituitary gland, both acting through their respective G-protein coupled receptor (GPCR). ACTH binds to the melanocortin-2 receptor (MC2R) in *zona fasciculata* cells, leading to the activation of adenylate cyclase, which ensures the conversion of adenosine triphosphate (ATP) to cyclic adenosine monophosphate (cAMP). cAMP activates its main intracellular mediators, EPAC (Exchange Protein Activated by cAMP), and a serine/threonine kinase called cAMP-dependent protein kinase A (PKA) (Bossis and Stratakis, 2004). The protein kinase A (PKA) holoenzyme is a heterotetramer that consists of two regulatory subunits each binding to one catalytic subunit (Bossis and Stratakis, 2004). Four regulatory (RIα, RIβ, RIIα, and RIIβ) and four catalytic (Cα, Cβ, Cγ, and Prkx) subunits have been described (Almeida and Stratakis, 2011). In order to activate PKA cAMP interacts with the regulatory subunit of PKA leading to a conformational change permitting the release of the catalytic subunits. The free catalytic subunits phosphorylate downstream targets such as cAMP response element-binding protein (CREB), which induce the transcription of target genes, such as those involved in cortisol synthesis (Figure 1) (Christenson et al., 1999; Manna et al., 2009). The intracellular cAMP is hydrolysed by specific phosphodiesterases (PDEs), and the two regulatory and catalytic subunits of PKA are reassembled in order to return to their inactive state (Stratakis and Boikos, 2007).

Several lines of evidence support cAMP's role in the development of cortisol-producing ACTs (Stratakis, 2014a). In McCune-Albright syndrome (MAS), which is caused by mutations in the *GNAS* gene that encodes the stimulatory subunit α of the G protein (Weinstein et al., 1991), ACAs or, more frequently, BAH are common. In CNC, inactivating mutations of the *PRKAR1A* gene (encoding the RIα subunit of PKA) (Kirschner et al., 2000a,b) lead to PPNAD. Mutations of the *PRKAR1A* gene have also been identified in sporadic cases of PPNAD (not associated with CNC), as well as in ACAs (Groussin et al., 2002; Bertherat et al., 2003). In addition, a number of *in vitro* and transgenic mouse studies have demonstrated that *PRKAR1A* is an adrenocortical tumor suppressor gene and its inactivation leads to ACTH-independent cortisol secretion (Sahut-Barnola et al., 2010; Almeida and Stratakis, 2011).

## *PRKACA* genetic defects lead to tumors of the adrenal cortex

In an initial cohort of 10 cortisol-producing ACAs associated with overt AICS, the Leu206Arg (c.617A>C) *PRKACA* recurrent mutation was identified in 70% of these cases; with one ACA having another *PRKACA* defect, Leu199_Cys200insTrp (Beuschlein et al., 2014). Both of these mutations affect residues that are highly conserved across species from invertebrates to humans suggesting the major role played by these amino acids is in protein function (Beuschlein et al., 2014; Goh et al., 2014). Dalmazi and collaborators have also identified two additional mutations: the insertion Cys200_Gly201insVal (c.600_601insGTG) and the missense Ser213Arg (c.639C>G) associated with 12 base pair duplication Leu212_Lys214insIleIleLeuArg (c.638_640insATTATCCTGAGG), in respectively 13.4% (3/22) and 4.5% (1/22) of their cortisol-producing ACA cohort (Di Dalmazi et al., 2014b). Recently, four independent projects of exome sequencing of cortisol-producing ACA development (Cao et al., 2014; Goh et al., 2014; Kubota et al., 2014; Sato et al., 2014) led to confirmation of *PRKACA*'s role in the pathogenesis of this neoplasm. The Leu206Arg variant has been identified at a frequency that ranges from 14.2 to 65.5% of cortisol-producing ACA depending on the studies (Beuschlein et al., 2014; Cao et al., 2014; Di Dalmazi et al., 2014a; Goh et al., 2014; Nakajima et al., 2014; Sato et al., 2014). No *PRKACA* mutations were found either in 1600 in-house exomes or in the 1000 Genomes Project data set or in blood of patients harboring *PRKACA* mutations in tumors. The Leu206Arg substitution likely alters the function of the Cα subunit at the heterozygote state, since both the wild type and mutant alleles were expressed in the tumor tissue (Beuschlein et al., 2014; Goh et al., 2014).

### Functional analysis of *PRKACA* mutations

*PRKACA* encodes the most highly expressed catalytic PKA isoform in the human adrenal and the functional consequences of two mutant variants have been predicted using different modeling approaches based on mouse PKA crystal structure (Beuschlein et al., 2014; Cao et al., 2014; Goh et al., 2014; Sato et al., 2014). In the absence of cAMP, the regulatory subunit fits into a highly conserved hydrophobic cleft in the catalytic subunit formed by Leu206 and Leu199. Therefore, the substitution from the small hydrophobic leucine to a large positively charge hydrophilic arginine in position 206 should abolish the interaction between the catalytic and regulatory subunit leading then to cAMP-independent PKA activation (Figure 1) (Beuschlein et al., 2014; Cao et al., 2014; Goh et al., 2014; Sato et al., 2014). Similar consequences are predicted for each one of the pathogenic *PRKACA* variants that have been identified so far in ACAs (Di Dalmazi et al., 2014a).

The activating effect of the novel *PRKACA* mutations in ACA predicted by what is known about the structural biology of the PKA tetramer has been validated by *in vitro* experiments in HEK293 cells for the most frequent variant, Leu206Arg (Beuschlein et al., 2014; Cao et al., 2014; Goh et al., 2014; Sato et al., 2014). The expression of Leu206Arg *PRKACA* increases the PKA activity and the level of CREB phosphorylation at Ser133 in basal conditions compared to the wild type *PRKACA* in two independent studies; the Leu206Arg did not interfere with the catalytic activity (Beuschlein et al., 2014; Cao et al., 2014; Goh et al., 2014). In contrast with cells transfected with the wild-type sequence, the Leu206Arg is not responsive to cAMP stimulation and its activity is not reduced by the co-expression with excess wild-type regulatory subunit (Beuschlein et al., 2014). The absence of interaction between this variant and the regulatory subunit was confirmed by FRET and co-immunoprecipitation experiments (Beuschlein et al., 2014; Goh et al., 2014; Sato et al., 2014). Altogether, these results demonstrated that the Leu206Arg mutant protein is constitutively active. Consistent with these conclusions, basal PKA activity in ACA with *PRKACA* mutations compared to those without mutations is increased (Beuschlein et al., 2014; Cao et al., 2014). Similarly, Goh and collaborators demonstrated by immunohistochemistry a higher staining of the phosphorylation of CREB at Ser133 in 8 *PRKACA* mutant ACA vs. 5 ACA without identified mutations (Goh et al., 2014). However, Sato and collaborators did not find any differences in phosphorylation level of CREB by Western blot (Sato et al., 2014).

### Clinical phenotype associated with *PRKACA* mutations

In total, the *PRKACA* gene has now been sequenced in 854 ACTs and no mutations have been found in cortisol-producing ACCs, non-secreting ACAs, androgen-secreting ACAs, aldosterone-producing ACAs, and adrenal oncocytomas. Thus, the overall frequency of the *PRKACA* hotspot mutation is 38.2% and it has been identified in cortisol-producing adenomas only (Table 1). One study described a predominance of *PRKACA* mutations in females (Cao et al., 2014). Patients harboring tumors with *PRKACA* mutations were diagnosed with CS at younger ages (45.3 ± 13.5 vs. 52.5 ± 11.9 years) (Goh et al., 2014). In five studies including both overt and subclinical CS (Beuschlein et al., 2014; Di Dalmazi et al., 2014b; Goh et al., 2014; Nakajima et al., 2014; Sato et al., 2014), *PRKACA* mutations are significantly associated with overt CS and higher serum cortisol level after 1 mg of dexamethasone, increased urinary free cortisol and midnight cortisol levels compared to patients without mutations (Beuschlein et al., 2014). These results highlight a direct link between *PRKACA* mutations and cortisol production, which is expected knowing the physiological function of PKA. In accordance with this observation, no *PRKACA* mutations have been found in subclinical CS patients in three independent studies (Beuschlein et al., 2014; Di Dalmazi et al., 2014b; Nakajima et al., 2014). However, Gao et al. and Sato et al. have found 11% (3/27) and 22% (2/9) of *PRKACA* mutations, respectively, at position 206 in subclinical CS patients (Table 1) (Goh et al., 2014; Sato et al., 2014). The term subclinical CS is used to describe cortisol-secreting tumors in patients without any typical symptoms of CS. However, its usage can vary between investigators and countries; this may explain the differences observed between these studies. Interestingly, in transcriptomic data from 25 wild-type ACA and 11 mutant ACA, 232 genes are differentially expressed and pathway analysis demonstrates an enrichment in “biosynthesis and metabolism of steroid and cholesterol” and “response to chemical stimulus” (Cao et al., 2014). This is in accordance with the essential role of PKA in the control of cortisol secretion.

**Table 1:** **Frequency of copy number gain (CNG) including** ***PRKACA*** **gene and** ***PRKACA*** **mutations in adrenocortical tumors**.

**References**	**Cortisol-producing ACA**	**Cortisol-producing ACA with over CS**	**Cortisol-producing BAH**	**Cortisol-producing ACC**	**Non-secreting ACA**	**Aldosterone-producing ACA**	**Androgen-producing ACA**	**Adrenocortical oncocytoma**
Beuschlein et al., 2014	22.2% mutations (22/99)	37% mutations (22/59^a^)	1.75% CNG (5/35^b^)	0% (0/42)	0% (0/20)	0% (0/20)	–	–
Cao et al., 2014	65.5% p.Leu206Arg (57/87)	NA	0% (0/13 PMAH)	0% (0/16)	–	–	–	0% (0/3)
Goh et al., 2014	23.6% p.Leu206Arg (57/87)	35% p.Leu206Arg (10/28)	–	0% (0/8)	–	–	–	–
Di Dalmazi et al., 2014b	32.3% mutations (22/68^c^)	34.3% mutations (22/64^c^)	0% (0/8)	0% (0/5)	–	–	–	–
Nakajima et al., 2014	14.2% p.Leu206Arg (3/21)	23% p.Leu206Arg (3/13)	–	–	0% (0/32)	–	0% (0/4)	–
Sato et al., 2014	52.3% p.Leu206Arg (34/65)	57.1% p.Leu206Arg (32/56)	–	–	–	–	–	–
Total	38.2% (151/395)	40%(89/220)	8.9% CNG (5/56)	0% (0/71)	0% (0/52)	0% (0/53)	0% (0/4)	0% (0/3)

a *21/59 harboring p.Leu206Arg and 1/59 with Leu199_Cys200insTrp*.

b *31 PPNAD + 2 iMAD+ 2 PMAH*.

c 18/22 harboring p.Leu206Arg; 3/22 Cys200_Gly201insVal; 1/22 Ser213Arg+Leu212_Lys214insIle-Ile-Leu-Arg

### *PRKACA* and other defects in ACAs

Altogether these results demonstrate that *PRKACA* mutations constitutively activate PKA leading to cortisol-producing adenomas, thereby suggesting that *PRKACA* is a main contributor to adrenocortical tumorigenesis. By whole-exome sequencing analysis, even if the number of exonic mutations is generally low, *PRKACA* is not the only oncogene mutated in cortisol-producing tumors. Activating mutations have also been identified in *CTNNB1* and *GNAS* genes at lower frequency (Cao et al., 2014; Goh et al., 2014; Sato et al., 2014). Importantly, *PRKACA* mutations were never found in association with other mutations. This suggests that the identified activating mutations may be mutually exclusive confirming the driver role played by Cα subunits in the development of cortisol-producing ACA. Interestingly, Goh and collaborators sequenced both ACA and ACC and were able to divide the ACA into two groups based on genetic results (Goh et al., 2014). Five out of 22 ACA are genetically closer to 3 ACC even if their Weiss score is 0 or 1, without any histological evidence of carcinoma. These findings are consistent with a progressive model of tumors forming in the adrenal cortex in the sequence hyperplasia-adenoma-carcinoma (Berthon et al., 2010; Stratakis, 2014b). However, the second ACA group, which included the ACA with *PRKACA* mutations, appears to have a distinct tumorigenesis mechanism. This is supported by the observation of three studies that tumors with mutations in *PRKACA* were significantly smaller than the non-mutant ones (Di Dalmazi et al., 2014b; Goh et al., 2014; Sato et al., 2014). Similarly, the weight of the sporadic ACT harboring *PRKAR1A* mutations is lower (11.2 ± 0.8 vs. 23.4 ± 12.05 g) (Bertherat et al., 2003). Therefore, PKA activation through constitutive activation of Cα subunit or RIα loss-of-function drastically increases cortisol secretion but has a limited impact on cell proliferation and tumor growth. Most recently, genomic duplication of the locus of *PRKACB* encoding for the Cβ catalytic subunit have also been described in a patient with CNC without CS (Forlino et al., 2014). It is possible that this reflects different roles of the two main catalytic subunits of PKA, with regards to their function in the adrenal cortex.

### *PRKACA* copy number gain and bilateral adrenocortical hyperplasia

Comparative genomic hybridization of 35 BAH with overt CS demonstrated copy number gain at chr19p locus that included *PRKACA* gene in 5 patients (Beuschlein et al., 2014; Stratakis, 2014a; Carney et al., 2015). The defect was present in the germline and there were no *PRKACA* coding sequence mutations. Two patients, a mother and a son, included in this study the same duplication and both presented with BAH (Beuschlein et al., 2014; Carney et al., 2015); this is the only case of inheritance in the cohort. Other duplications at this locus have not been found in 24 cortisol-producing ACAs (Carney et al., 2015). Interestingly, the histological phenotype of these five patients has been published and 3 of them looked like PPNAD. This phenotype is comparable with PKA activation through *PRKAR1A* inactivation or PDEs mutations causing PPNAD (Almeida and Stratakis, 2010). However, the two remaining patients did not have PPNAD but diffuse adrenal cortex hyperplasia with nodules (Carney et al., 2015). This demonstrates that the same genetic alteration can lead to different histological phenotypes. It also demonstrates that differences in *PRKACA* gains and, thus, functional “dosage” have different effects on the histology of the adrenal cortex (Stratakis, 2014a). Whereas somatic mutations that lead to overactivity cause ACAs (Stratakis, 2014a), germline defects cause BAH depending on genetic dosage (Lodish et al., 2015).

### Is Cα a new therapeutic target for cushing syndrome?

*PRKACA* mutations have been described in almost 40% of cortisol-producing ACA and are therefore, the most frequent genetic alteration in these tumors (Table 1). Beyond the importance of PKA in adrenal, its involvement in genetic diseases like CNC and cancers has been demonstrated (Caretta and Mucignat-Caretta, 2011). The ability to inhibit its constitutive activation through chemical components is a major challenge due to its critical role on cell function. Two inhibitors H89 and KT5720 have successfully decreased PKA activity induced by the transfection of Leu206Arg variant in HEK293 cells (Sato et al., 2014). These two inhibitors have been extensively used to better understand the role of PKA, however, their lack of specificity has been well established (Lochner and Moolman, 2006; Murray, 2008). Most of the inhibitors targeted the ATP-binding site but this was problematic due to the high percentage of identity of this domain among the Ser/Thr kinases family responsible to the low specificity of these inhibitors (Sapio et al., 2014). Moreover, these inhibitors cannot be used for activating *PRKACA* mutations. Better understanding of PKA function permits the development of substrate-competitive inhibitors, which would be more specific as there is diverse substrate-binding domain. The PKA inhibitor (PKI) is an endogenous thermostable peptide that interacts with the catalytic domain and is able to inhibit Leu206Arg variant *in vitro* (Cao et al., 2014). However, its main disadvantage preventing its use for clinical application is its weak permeability and its susceptibility to proteases. The discovery of new PKA inhibitors is desirable and can be helpful in the treatment of cortisol-producing ACA but also others cancers.

## Conclusions

The discovery of somatic mutations in *PRKACA* is one additional proof of the central role of cAMP-PKA pathway in the development of cortisol-producing ACA. The high frequency of mutations (approximately 40%) and the even higher presence of PKA function alterations (Bimpaki et al., 2009), suggest that perhaps other components of the pathway may also be found mutated in the future. However, as *PRKACA* is ubiquitously expressed, its mutations may also be found in other tissues with PKA-dependent tumorigenesis. Future analysis and use of animal models will provide useful information to help answer this, and other, questions.

### Conflict of interest statement

The authors declare that the research was conducted in the absence of any commercial or financial relationships that could be construed as a potential conflict of interest.
